# Regulatory Immune Cells in Idiopathic Pulmonary Fibrosis: Friends or Foes?

**DOI:** 10.3389/fimmu.2021.663203

**Published:** 2021-04-22

**Authors:** Chiel van Geffen, Astrid Deißler, Markus Quante, Harald Renz, Dominik Hartl, Saeed Kolahian

**Affiliations:** ^1^ Department of Experimental and Clinical Pharmacology and Pharmacogenomics, University Hospital Tübingen, Tübingen, Germany; ^2^ Department of General, Visceral and Transplant Surgery, University Hospital Tübingen, Tübingen, Germany; ^3^ Institute of Laboratory Medicine and Pathobiochemistry, Molecular Diagnostics, Philipps University of Marburg, Marburg, Germany; ^4^ Universities of Giessen and Marburg Lung Center, German Center for Lung Research (DZL), Marburg, Germany; ^5^ Department of Pediatrics I, Eberhard Karls University of Tübingen, Tübingen, Germany; ^6^ Dominik Hartl, Novartis Institutes for BioMedical Research, Basel, Switzerland

**Keywords:** idiopathic pulmonary fibrosis, mesenchymal stem/stromal cells, regulatory T cells, regulatory B cells, macrophages, myeloid-derived suppressor cells, pharmacotherapy, transplantation

## Abstract

The immune system is receiving increasing attention for interstitial lung diseases, as knowledge on its role in fibrosis development and response to therapies is expanding. Uncontrolled immune responses and unbalanced injury-inflammation-repair processes drive the initiation and progression of idiopathic pulmonary fibrosis. The regulatory immune system plays important roles in controlling pathogenic immune responses, regulating inflammation and modulating the transition of inflammation to fibrosis. This review aims to summarize and critically discuss the current knowledge on the potential role of regulatory immune cells, including mesenchymal stromal/stem cells, regulatory T cells, regulatory B cells, macrophages, dendritic cells and myeloid-derived suppressor cells in idiopathic pulmonary fibrosis. Furthermore, we review the emerging role of regulatory immune cells in anti-fibrotic therapy and lung transplantation. A comprehensive understanding of immune regulation could pave the way towards new therapeutic or preventive approaches in idiopathic pulmonary fibrosis.

## Introduction

Pulmonary fibrosis (PF) is a chronic lung disease characterized by progressive fibrotic tissue remodeling and scarring of lung tissue (1). Various factors, such as smoking, chronic aspiration due to gastroesophageal reflux, infections, toxins, radiation, autoimmune reactions (e.g. rheumatoid arthritis, scleroderma, polymyositis, dermatomyositis, Sjögren’s syndrome or systemic lupus erythematosus) and exposure to environmental pollutants can trigger chronic lung tissue damage resulting in fibrotic remodeling (2–6). However, PF can also occur without an identifiable underlying cause, known as idiopathic pulmonary fibrosis (IPF). IPF is an age-related interstitial lung disease, affecting mainly patients of 50 years and older with an incidence of 2.8–18 per 100,000 people and a prevalence of 1.25–27.9 per 100,000 people in Europe and North America (3). Unfortunately, IPF patients have a poor prognosis with a median survival of 2-4 years after diagnosis (3). A gain-of-function mutation in the *MUC5B* gene represents the highest genetic risk factor for the development of IPF (3). IPF is difficult to treat with pharmacological therapies and to date, the only effective curative therapy for IPF patients is lung transplantation (3).

Chronic alveolar-micro injuries presumably lead to a maintained and dysregulated wound healing process, which drives IPF (3). Fibrogenesis is marked by a massive accumulation of extracellular matrix (ECM) produced by myofibroblasts such as collagen, elastin, laminin, fibronectin, hyaluronan and glycoproteins, resulting in irreversible thickening of alveolar walls, compromising the exchange of oxygen and carbon dioxide between blood and alveolar air (7, 8). At the cellular level, repeating lung injuries mostly affect type I alveolar epithelial cells (AECs), which mainly form the alveolar surface. In response to this cell loss, type II AECs proliferate in a hyperplastic manner to mask the exposed basement membrane (2). Under healthy conditions, the cells would differentiate into type I AECs and hyperplastic type II AECs would undergo apoptosis (2). However, under the influence of transforming growth factor (TGF)-β, hyperplastic type II AECs remain at the alveolar surface resulting in alveolar collapse (2). Fibroblasts are the most frequent cell type in fibrotic tissues that produce ECM-producing cells which are recruited into the lung compartment (2). Fibroblasts differentiate into contractile myofibroblasts with massive ECM productive capacity (2). Cytokines and growth factors activating fibroblasts and myofibroblasts and inducing further fibrotic tissue remodeling include TGF-β, interleukin (IL)-1, IL-6, IL-13, IL-33, platelet-derived growth factor (PDGF), fibroblast growth factor (FGF), tumor necrosis factor (TNF)-α and leukotrienes (7). Activated fibroblasts produce TGF-β, IL-1β, IL-33, reactive oxygen species, C-X-C motif chemokines (CXC), C-C motif chemokines (CC) maintaining fibrogenesis and attracting immune cells to promote chronic inflammation, resulting in a positive feedback loop supporting fibrogenesis through differentiation of fibroblasts into myofibroblasts (7). TGF-β itself contributes to fibrosis progression *via* TGF-β/SMAD signaling by stimulation of ECM production, inhibition of ECM breakdown through matrix metalloproteinases (MMPs), and epithelial-mesenchymal transition (EMT) induction (9).

**Figure 1:**
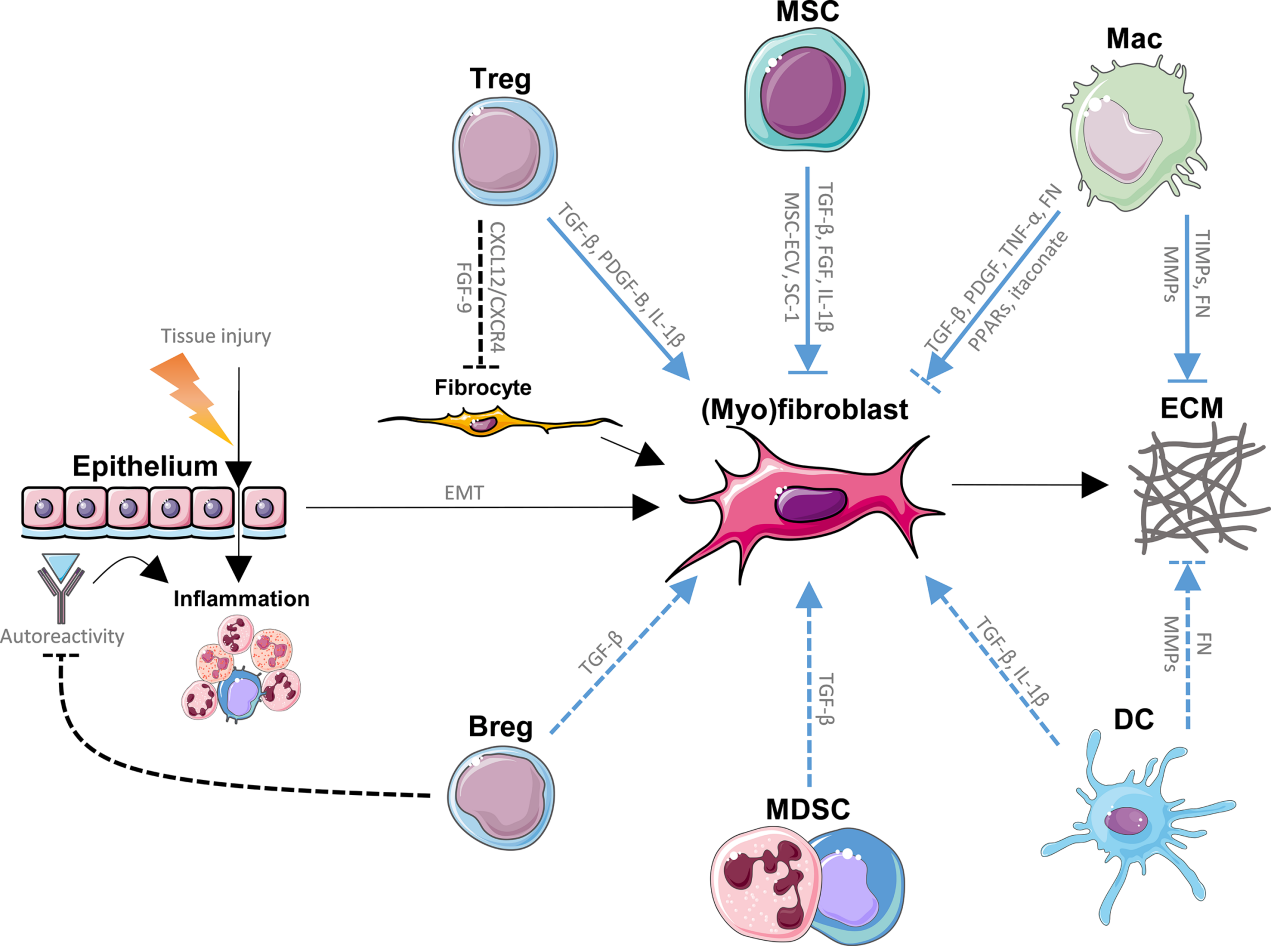
Direct effects of regulatory immune cells on (myo)fibroblasts and extracellular matrix (ECM) in idiopathic pulmonary fibrosis (IPF). Repeated tissue injury triggering chronic tissue damage resulting in inflammation, epithelial mesenchymal transition (EMT) and ultimately in excessive production and buildup of ECM by fibrocytes and (myo)fibroblasts (fibrosis) in the lungs, represents the main paradigm involved in the pathology of IPF. Immune cells with regulatory functions modulate (myo)fibroblast generation, (myo)fibroblast function and ECM homeostasis through various signaling pathways, and known direct pathways are listed here. Mesenchymal stem/stromal cells (MSCs) promote (myo)fibroblasts through fibroblast growth factor (FGF), transforming growth factor (TGF)-β and interleukin (IL)-1β, while MSC-derived extracellular vesicles (MSC-ECV) and stanniocalcin (SC)-1 have opposite effects. MSCs are also prone to myofibroblastic transition. Tregs promote fibrogenesis through TGF-β, platelet-derived growth factor (PDGF)-B and IL-1β, while inhibiting the recruitment of fibrocytes by inhibition of the CXCL12/CXCR4 axis as well as FGF-9. Macrophages enhance fibrosis through TGF-β, PDGF, tumor necrosis factor (TNF)-α, fibronectin (FN) and inhibit fibroblasts *via* itaconate and peroxisome proliferator-activated receptor (PPAR) ligands. Myeloid-derived suppressor cells (MDSCs) and regulatory B cells (Bregs) have been suggested to activate lung fibroblasts, possibly through TGF-β. Bregs inhibit autoreactive immunoglobulins, which may deposit in lung tissue and promote inflammation and IPF. Dendritic cells (DCs) have been shown to produce pro-fibrotic TGF-β and IL-1β. Macrophages (Macs) as well as DCs break down the ECM *via* matrix metalloproteinases (MMPs), a process that is inhibited by tissue inhibitors of metalloproteinases (TIMPs) produced by other macrophage subtypes. Both Macs and DCs have been found to produce fibronectin (FN), another ECM component. Blue lines represent the direct effects of regulatory immune cells on (myo)fibroblasts and ECM in IPF. Dashed lines represent interactions that are not firmly established in IPF. This figure was created using Servier Medical Art templates, which are licensed under a Creative Commons Attribution 3.0 Unported License; https://smart.servier.com.

Cells of both the innate and adaptive immune system such as mesenchymal stem/stromal cells (MSCs), regulatory T cells (Tregs), regulatory B cells (Bregs), macrophages, dendritic cells (DCs) and myeloid-derived suppressor cells (MDSCs) have been linked to the pathogenesis of IPF, often with contradicting findings (**Figure 1**) (10–14). Immunomodulation by regulatory immune cells is crucial in dampening pathogenic immune responses and inhibiting the transition from inflammation to fibrosis. Identifying the role of regulatory immune cells in IPF is therefore key in understanding the imbalanced immune responses underlying IPF. In this review, we summarize and critically discuss the role of regulatory immune cells in IPF, and assess their interaction with current pharmacological therapies and lung transplantation in IPF.

## Regulatory Immune Cells in IPF

### Mesenchymal Stem/Stromal Cells

MSCs are multipotent stem cells that can differentiate into various cell types, including osteoblasts, chondrocytes, adipocytes, myocytes, fibroblasts and endothelial cells (15). Furthermore, MSCs play an important role in modulating immunity (16). MSC phenotype and function are dependent on environmental cues (17). Lung-resident (LR)-MSCs have been proposed to play a role in lung regeneration. The expression of high levels of telomerase by LR-MSCs provides self-renewal, survival and replication capacity which may promote lung regeneration by repopulating structural lung cells (17). LR-MSCs have also been suggested to play an important role in IPF. Several profibrotic factors, as mentioned above, have been found to induce myofibroblastic transition (MFT) of MSCs, which in turn promotes collagen production and ECM accumulation driving PF (17–21). Bleomycin-induced pulmonary fibrosis (BPF) in mice resulted in the loss of LR-MSCs, likely due to MFT induced by the upregulation of previously mentioned profibrotic factors (22). Inhibition of the underlying signals mediating MFT, such as Hedgehog, Wnt/β-catenin, Shh/Gli – Wnt or nuclear factor (NF) - κB signaling, were shown to reduce myofibroblast differentiation of LR-MSCs and attenuated BPF in mice (18, 23–25). Dysregulations in these pathways have been found to modulate TGF-β, MMPs, ECM production and EMT (9, 26). Manipulating the MFT of LR-MSCs by regulating microRNA-497 expression understated their detrimental effect in BPF (27). The expression and activity of collagen prolyl hydroxylase, an enzyme crucial for collagen synthesis, by fibrotic lung mesenchymal cells was observed to be one of the mechanisms underlying the TGF-β-mediated profibrotic effects (28). Suppression of collagen prolyl hydroxylase by pyridine-2,5-dicarboxylate attenuated TGF-β-mediated collagen production in both cultured fibroblasts as well as in BPF (28). Promotion of REVERBα, a transcriptional repressor that is upregulated in IPF, has been shown to inhibit both myofibroblast differentiation and collagen secretion in organotypic cultures from IPF patients (29).

In contrast, there is an increasing scientific interest in MSCs due to their immunomodulatory and anti-inflammatory effects (15) as well as an increasing amount of evidence showing anti-fibrotic effects of MSCs in rodents. MSC therapy in BPF models in rodents showed reductions in lung collagen deposition (22, 30–43), lung fibrosis (22, 32, 34, 35, 37, 38), TGF-β levels (22, 30–36, 38–41) and total and/or neutrophil cell count in the bronchoalveolar lavage fluid (BALF) (34, 36, 37, 39) and improved 14-day survival (22, 34, 36) after bleomycin administration. A large body of research is focused on MSC therapies in a variety of human diseases, including graft versus host disease (GVHD), autoimmune disorders, acute respiratory distress syndrome (ARDS), chronic obstructive pulmonary disease (COPD) and asthma (44) as well as in novel diseases like COVID-19 (45, 46). Autologous or allogeneic MSCs are harvested from the peripheral blood, adipose tissue, bone marrow, umbilical cord, placenta, dental pulp, synovial fluid, endometrium, skin or muscle. Due to low numbers, harvested MSCs are usually expanded *in vitro* in culture medium supplemented with fetal bovine serum, before being transferred to patients (44). The use of MSC-derived products, such as MSC-derived vesicles and exosomes were also reported (47).

At the time of writing, there are five clinical trials using MSC therapy to treat IPF, as registered on the National Institute of Health (NIH) clinical trial database (48)^1^. A phase Ib clinical trial, completed in 2015, studied the intravenous administration of 1 to 2*10^6^ MSC/kg allogeneic placenta-derived MSCs in patients with moderately severe IPF (NCT01385644) (49). The phase I AETHER trial, completed in 2016, studied the administration of a single intravenous infusion of 20, 100, or 200*10^6^ allogeneic bone marrow-derived MSCs in patients with mild-to-moderate IPF (NCT02013700) (50). Another phase I clinical trial, completed in 2018, studied the safety and feasibility of endobronchial administrated autologous bone marrow-derived MSCs in patients with mild-to-moderate IPF (NCT01919827). An uncompleted phase I/II clinical trial that planned to study adipose-derived MSCs in IPF has not recently been updated on its status (NCT02135380). Finally, a phase I/II clinical trial, completed in 2018, studied the intravenous administration of two doses of 2*10^8^ allogeneic bone marrow-derived MSCs every 3 months for a total of 1.6*10^9^ MSCs in patients with moderate-to-severe IPF (NCT02594839) (51). Another phase Ib clinical trial studied the safety of 1.5*10^6^ autologous adipose-derived MSCs-stromal vascular fraction/kg infused into the lungs of patients with mild-to-moderate IPF at monthly intervals (52). Four of the completed clinical trials published their results showing that MSC therapy is a safe, feasible and promising method to treat IPF patients (49–52). However, more clinical trials are necessary to prove the efficacy of MSC therapy in IPF patients. Due to the urgency and severity of many diseases, including IPF, there are many reports of unapproved stem cell therapies being used, often due to a lack of regulation (53, 54). There remain key challenges in MSC therapy, such as identifying the most valuable source of MSCs, the ideal donors and recipients and the most effective handling and administration routes. Other challenges are in identifying during which stages of IPF patients are most susceptible to MSC therapy as well as possible interactions with pharmaceuticals.

Beneficial effects of MSC therapy in IPF have been suggested to be modulated by MSCs promoting alveolar repair by the secretion of growth factors (55), suppressing inflammation by the production of nitric oxide (NO) and indoleamine 2,3-dioxygenase (IDO) (56), promotion of Treg expansion (57), decreasing pro-inflammatory cytokines such as TNF-α, interferon (IFN)-γ and IL-2 by secreting IL-10 and soluble IL-1β receptor (56) and by protecting lung injury by restituting alveolar bioenergetics through mediating mitochondrial transfer (58). Other observed anti-fibrotic pathways were the production of stanniocalcin-1 which is shown to dampen BPF in mice (41), MSC-derived extracellular vesicles which were found to suppress TGF-β1-induced MFT (47, 59, 60), and MSCs ability to differentiate into type II lung cells expressing surfactant (60). MSCs are also immunoprivileged, lacking human leukocyte antigen (HLA) class II combined with low HLA class I expression, allowing the administration to immunocompetent patients without the need for further immunosuppression (61). However, transferred MSCs have been shown to secrete pro-fibrotic TGF-β1 and underwent MFT in BPF in mice (57). In contrast, MSCs secreting higher levels of TGF-β1 have been suggested to have optimal anti-fibrotic efficacy in BPF in mice (57).

Recent evidence suggests an increase in senescent bone marrow-derived (BM-) MSCs (expressing the senescence markers p21, p16^INK4A^, p53 and senescence-associated β-galactosidase) in IPF patients contribute to disease progression (62, 63). Accumulation of DNA damage in senescent IPF BM-MSCs resulted in decreased mitochondrial and cellular function (62). These findings are in line with the observed decrease in migration capacity and reduced anti-fibrotic effects of BM-MSCs derived from IPF patients, which were adoptively transferred in BPF mice (62). Administrated MSCs derived from older mice were found to be less successful in ameliorating BPF compared to those derived from younger rodents, supporting the contribution of MSC senescence (64). DNA protein kinase catalytic subunits, involved in DNA repair mechanisms, were found to be reduced in IPF lung tissue, which was suggested to result in the expansion of stage-specific embryonic antigen-4^+^ mesenchymal progenitors, and the senescence of mesenchymal progeny (63). The possible involvement of senescent MSCs in IPF disease progression may be correlated with age being the most important risk factor (62).

Taken together, literature suggests that an increase in MFT of LR-MSCs as well as an increase in MSC senescence are detrimental to IPF progression. However, the immune regulatory role of MSC has been linked with beneficial effects in IPF, and, as evidenced by the positive findings of studies investigating MSC therapy, seem to provide promising treatment strategies.

### Regulatory T cells

Tregs have been found crucial in modulating immunity and maintaining immune tolerance, but their role in IPF remains contradicting and unclearly defined (65, 66). Activated CD4^+^ T cells (67–69) as well as Tregs (70–73) were found to be upregulated in the peripheral blood of IPF patients and positively correlated with disease severity (67, 68, 70, 71, 73). Tregs were recruited to the lungs of murine models of PF (74, 75). In contrast, several studies observed reduced Treg numbers with reduced suppressive functions in the peripheral blood and BALF of IPF patients correlating to disease severity (76, 77).

Tregs have been suggested to be able to protect from TGF-β1-induced fibrosis *via* the release of IL-10, as Th1 cells exposed to TGF-β1 produce IL-10 (78). TGF-1β was shown to ameliorate BPF in wild-type mice, but not in IL-10-deficient mice (78). IL-10 also has been found to down-regulate type I collagen synthesis in human scar tissue-derived fibroblasts (79) and protect against BPF (80). However, TGF-β1 may also mediate the potential negative role that Tregs play in IPF (81). Tregs have been shown to increase collagen deposition through TGF-β1 in fibroblasts (82) and in lymphatic tissue (83) as well as in the lungs (74) of mice. TGF-β1 autocrine signaling in Tregs was also shown to induce secretion of pro-fibrotic PDGF-B in non-inflammatory conditions (74). Early depletion of Tregs indeed reduced TGF-β levels and lung fibrosis in BPF in mice (81). Semaphorin 7A is an immunomodulating protein involved in many processes such as monocyte chemotaxis (84) and DC migration (85). Adoptive transfer of semaphorin 7A-positive Tregs was found to exaggerate TGF-β1-induced lung fibrosis in TGF-β1-transgenic mice (70). However, the regulation of TGF-β1 levels is not limited to Tregs, confirmed by the finding that reduced Tregs and Foxp3 expression in mice lacking phosphatase and tensin homolog (an antagonist of phosphatidylinositol 3-kinases, which mediate myeloid effector function) was combined with a massive upregulation of TGF-β1 in the lungs (86).

The importance of the CXCL12/CXCR4 axis in IPF has previously been described, and is thought to contribute to fibrosis through recruitment and modulation of fibrocytes (77, 87, 88). Fibrocytes are cells expressing CD45, collagen-1 and CXCR4, exhibit pro-inflammatory and ECM-remodeling properties and share key features with fibroblasts (89, 90). Indeed, the recruitment of CXCR4^+^ fibrocytes to the lungs of IPF patients was confirmed and blockage of CXCR4 ameliorated BPF in mice (87, 88). Tregs were found to decrease CXCL12 expression and may therefore play a key role in reducing fibrocyte recruitment (91). Similarly, Tregs have been found to inhibit fibrocyte recruitment *via* suppression of FGF-9 (92).

Another pathway by which Tregs may influence IPF progression is by mediating the balance between T helper (Th) 1, Th2, and Th17 responses. Th1 cells were shown to exhibit anti-fibrotic effects through the production of IFN-γ and IL-12 (93), and IFN-γ production by T cells was found to be suppressed by Tregs (94). Th2 immune responses were shown to stimulate myofibroblasts and the progression of the disease through the production of IL-4, IL-5, IL-13 and IL-17A (95, 96). In radiation-induced lung disease in mice, an increase in Th17 was linked to enhanced lung fibrosis, while an increase in Th1 was found to have the opposite effect (97). The downregulation and depletion of Tregs as well as the neutralization of Treg-immunosuppressive activity has been shown to switch Th2-driven responses to Th1-driven responses (74, 98, 99) and attenuated PF progression in a silica-induced (99) as well as in an irradiation-induced murine model (100). Tregs were suggested to promote Th17 through TGF-β and IL-1β (100, 101). However, Th17 cells were found to be decreased in the peripheral blood (while Tregs were found upregulated) resulting in increased TGF-β/IL-17 ratio in IPF patients (72). Adoptive transfer of Tregs into recombination activating gene (Rag) 2 protein-deficient mice (lacking lymphocytes) was found to promote BPF progression and a loss of Foxp3 expression after transfer (10, 102). These findings suggest Tregs might lose their suppressor ability and adopt a Th2 phenotype through which they may promote disease progression (10, 102). In contrast, pro-inflammatory CD4^+^CD28^null^ helper T cells were found to be upregulated in IPF and were found to be unresponsive to Treg immunomodulation (103).

Treg expansion using IL-2 therapies was shown successful in treating diseases like vasculitis and GVHD (104, 105). Soluble IL-2 complexed to an IL-2 receptor neutralizing antibody allows for hyperstimulation of CD25 (IL-2 receptor alpha chain) by depleting the IL-2 receptor, which in turn promotes Treg expansion. Administration of IL-2 complex in a mouse model of BPF was found to exacerbate PF by increasing collagen deposition as well as inducing lung remodeling (102). The immune response was found to be switched from a Th1 to a Th2 response, while TGF-β production remained unaltered (102).

The adoptive transfer of Tregs did show positive effects in mice models of acute lung injury (91, 106). Adoptive transfer of Tregs was observed to reduce delayed lung recovery in Rag1^-/-^ mice (lacking mature T and B cells) (106) as well as fibroproliferation (91) in lipopolysaccharide-induced acute lung injury. Interestingly, while early depletion of Tregs was found to reduce lung fibrosis and TGF-β1 expression in BPF in mice, late depletion of Tregs showed opposite effects (81). Late stage (after 21 days) increase of Tregs in BPF in CC receptor (CCR) 7 (receptor involved in B and T cell activation, T cell homing and DC maturation) deficient mice showed a positive suppressive effect of Tregs in IPF as well (107). These results suggest several translational limitations relating to animal models of PF that likely resulted in several contradicting results regarding the role of Tregs in IPF (10). Commonly used animal models, such as BPF, are not specific to IPF and rather poorly replicate the core characteristics of the disease, such as the slow development and its irreversible nature. Combined with the already existing gap between animals and humans, as well as the complexity of Tregs, it further complicates translation to the clinical setting.

Literature on the role of Tregs in IPF is often contradictory and the explanation to this phenomenon may be hiding in several factors (10). First of all, the phase of IPF progression appears to be of vital importance when assessing the role of Tregs. Second, there seems to be a high likelihood of discrepancies in the number of Tregs found in the periphery (i.e. the blood) versus the number of Tregs observed in the lungs. Third, there are translational limitations relating to animal models of IPF. Combined with the diverse role of Tregs, it is reasonable to assume these factors contributed to the contradicting observations regarding their role in IPF. All in all, Tregs appear to be both friend and foe in relation to IPF, which suggests that Treg modulation requires careful assessment of the phase of IPF disease state.

### Regulatory B Cells

B cells mediate the humoral immunity of the adaptive immune system by secreting antibodies (as plasma cells), and mediate immunity by functioning as antigen-presenting cells (APCs) and by producing cytokines (108). The amount of circulating B cells in the peripheral blood (109, 110) and B cells in the lungs (110–114) of IPF patients were found to be increased together with CXCL 13 (a critical chemokine for the homing of B cells) (115, 116), correlating with disease progression. Similarly, there was an increase in IL-6, IL-13 and B-cell-activating factor (BAFF) (all factors that promote B cells) found in IPF patients (110). Rag1^-/-^ mice as well as gp130(757F);uMT^-/-^ compound mutant mice (deprived of mature B cells) were protected from BPF, suggesting a negative role of B cells in IPF progression (117).

Bregs, however, have been shown to dampen T-cell driven immune responses, to support immunological tolerance, and have been shown to produce anti-inflammatory IL-10 and TGF-β (118). A loss of Bregs in both allergic and autoimmune diseases was found to exacerbate disease progression (119). Decreased numbers of Bregs were found in IPF patients, as well as an increase in the proliferation and activation of T follicular helper (Tfh) cells (12). Tfh cells are a specialized Th cell subset that assist B cells by the secretion of IL-4, IL-10 and IL-21 and CD40 ligand expression, which promotes the growth, differentiation and initiates class switching of B cells as well as induce antigen-specific antibody responses (120). Bregs were shown to suppress Tfh-cell maturation and inhibit Tfh cell-mediated antibody secretion (121). Increases of circulating antigen differentiated B cells (110), plasmablasts (110), autoreactive immunoglobulin (Ig) A in the lungs (109), Ig in BALF (122) and immune complexes were observed in the BALF and lungs of IPF patients (123–126). These findings are in line with the observed reduction of Bregs and observed increase of both Tfh and non-regulatory B cells in IPF. Autoreactive immune complexes are able to deposit in the lung and promote inflammation, while antibodies mediate antibody-dependent cellular cytotoxicity (mediated *via* complement activation or natural killer cells), processes that may drive IPF progression (127).

Antibodies, such as Rituximab, target the CD20 marker that is expressed on the surface of all B cells starting at the pre-B cell stage (128). These antibodies therefore inhibit CD20^+^ B cells, which as described above were found to be positively correlated to IPF progression (109–117). Rituximab indeed may be a viable treatment option of IPF (129, 130), and is included in 6 NIH registered clinical trials treating IPF at the time of writing (48)^2^. Unfortunately, CD20 antibodies also target CD20 expressing Bregs, showing a downside to unspecific downregulation of B cells. However, levels of CD20 expression differs among different B cells populations, with Bregs generally expressing lower levels of CD20 (131). Depletion of most CD20^+^ B cells by rituximab was found to enrich CD20^low^ Bregs in mice and human cancer (131). The specific targeting of Bregs or non-regulatory B cells is limited by the lack of specific surface markers (132). However, Bregs might be targeted and upregulated *via* other pathways such as immunomodulation by MDSC (133). A study by Wu et al. found that PGE2 in the exosomes derived from polymorphonuclear (PMN)- MDSCs promote IL-10^+^ B cells and ameliorates collagen-induced arthritis in mice (133).

Collectively, Bregs seem to be able to play a vital role in the suppression of IPF. Progress in the specific depletion of non-regulatory B cells or in the expansion of Bregs may provide promising clues for future treatment strategies.

### Macrophages

Macrophages play an important role in mediating tissue homeostasis and inflammation as well as phagocytosing viral, bacterial and parasitic pathogens and inducing the adaptive immune response by functioning as an APC (134). The disturbed balance in wound healing processes underlying IPF is thought to be primarily mediated by macrophages (11). Furthermore, the interplay of MMPs and tissue inhibitors of metalloproteinase (TIMPs), secreted by macrophages, among other secreted factors, such as collagen, shape the ECM. Macrophages thereby contribute to IPF in either a pro- or anti-fibrotic manner (135).

Increased number of macrophages was observed in the lungs of IPF patients (136), yet their functional role remains a matter of active discussion. Lung macrophages have been shown to play a crucial role during the fibrotic phase of BPF in mice (136). Low numbers of macrophages during the resolution phase were found to reduce ECM degradation and exacerbate PF (137). Depletion of macrophages in an early phase of fibrosis was, however, shown protective in a murine model of liver fibrosis (137). Previously, the M1/M2 macrophage polarization nomenclature, inspired by the Th1/Th2 paradigm, was commonly used to classify macrophages (86, 96, 138–149). M1-like macrophages have been found to promote fibrosis by secreting pro-inflammatory IL-6 and TNF-α as well as promoting the Th17 and neutrophilic immune response (86, 139–141). M2-like macrophages have been linked to fibrotic processes through the promotion of CC ligand (CCL) 2 and 17, TIMPs, fibronectin, Th2 response through IL-4 production, fibroblasts and the MFT of LR-MSCs (96, 142–149). On the other hand, the production of arginase-1 and MMPs, as well as anti-inflammatory IL-10, TGF-β1 and heme oxygenase 1 by M2-like macrophages suggest a protective role in IPF (96, 142, 143, 149). Peroxisome proliferator-activated receptor (PPAR-) α and PPAR-γ activation has been found to inhibit inflammation by inducing M2-like macrophages, which in turn may inhibit pro-inflammatory cytokine production (150, 151). These findings suggest PPAR ligands may be interesting therapeutic targets in IPF, especially considering they have also been found to directly inhibit fibroblast activation induced by TGF-β1 (152). However, as the M1/M2 paradigm is based on findings from *ex vivo* cultured cells, it is unable to accurately recapitulate the complexity of macrophage phenotypes *in vivo*, and, especially those observed in the IPF lung (153).

Recent studies have begun profiling individual cells, including macrophages, in IPF in great detail through single-cell RNA sequencing (scRNA-seq) (154). Several research groups made their scRNA-seq findings on thousands of cells derived from the lungs of IPF and healthy control available (154). Analysis of such data sets enables the identification of many novel macrophage subsets and their specific ontogeny relating to different stages of IPF. Highly proliferative osteopontin-expressing macrophages were identified through scRNA-seq and were found to mediate myofibroblast activation in the lungs of IPF patients (155). Similarly, another expanded population of pro-fibrotic CD36^+^CD84^+^ macrophages in IPF were identified, which also showed increased expression of genes involved in ECM remodeling (156, PREPRINT).

On the other hand, itaconate has recently been described to mediate anti-fibrotic effects of macrophages in the lungs (157). Decreased levels of pulmonary itaconate as well as reduced itaconate-synthesizing cis-aconitate decarboxylase (*ACOD1*) expression (which mediates itaconate production) in lung macrophages were observed in IPF patients (157). *Acod1* deficient mice developed more severe BPF, while adoptive transfer of lung-derived monocytic macrophages from healthy donor mice alleviated the developed persistent fibrosis (157). Furthermore, itaconate was shown to decrease fibroblast proliferation and wound healing capacity of cultured fibroblasts (157).

The pentraxin-2 analogue, PRM-151, which inhibits the differentiation of monocytes into pro-inflammatory and pro-fibrotic macrophages (and fibrocytes), is therefore a promising therapy and is currently undergoing clinical trial for the treatment of IPF (158). However, several studies suggest possible anti-fibrotic and immunomodulating roles of macrophages, which may be suppressed in IPF, and therefore might provide interesting therapeutic targets. Furthermore, the recent unraveling of specialized macrophage subsets through tools such as scRNA-seq is rapidly increasing the understanding of the complex role they play in IPF and is expected to accelerate future research development substantially.

### Dendritic Cells

DCs bridge the innate and adaptive immunity through their role as APCs (159). Besides their primary role as immune activators, DCs have also been described to play tolerogenic and regulatory roles (13). These tolerogenic DCs are crucial in the maintenance of central and peripheral tolerance by inducing clonal T cell deletion, T cell anergy, inhibition of memory and effector T cell responses and promoting Tregs (160).

DCs accumulate in the fibrotic lungs of BPF in mice (161, 162) as well as in the BALF and lungs of IPF patients (163–165), while circulating DCs were decreased (166). Fms-like tyrosine kinase-3 ligand (Flt3L), a cytokine that promotes differentiation and proliferation of DCs, was found to be upregulated in the peripheral blood and lungs together with an increase in DCs in the lungs of TGF-β1-induced murine model of PF as well as in IPF patients (167). The unspecific deprivation of DCs in mice lacking Flt3L resulted in more severe PF, while unspecific upregulation of DCs, by supplementing Flt3L, resulted in reduced PF progression in a TGF-β1-induced PF mouse model (167). Specific depletion of DCs by diphtheria toxin (DT) in a mouse strain expressing zinc-finger and BTB domain containing 46 (*Zbtb46*; transcription factor keeping DCs in a quiescent state)-DT receptors was found to result in severe PF in a TGF-β1-induced mouse model of PF (167). In contrast, depletion of DCs by the administration of DT to CD11c-DT receptor-transgenic mice attenuated BPF (168). However, in the latter study it is important to note that CD11c-expressing macrophages were depleted as well, which may explain the discrepancy between the two studies (168). Inactivating DCs using VAG539, a pro-drug of VAF347, which activates the transcription factor aryl hydrocarbon, attenuated BPF lung injury in mice (162). However, VAG539 modulates the cell physiology by binding the transcription factor aryl hydrocarbon receptor expressed in many other cell types besides DCs (169), and has been shown to promote the development of IL-22-secreting Th cells (170), making it questionable whether the attenuation can be attributed solely to DC inactivation. CD11b^+^ DCs have been found to upregulate the expression of several MMPs, promoting collagen and ECM degradation (171). CXCL4 has recently been identified to be crucial in altering DC development into a pro-inflammatory and pro-fibrotic phenotype that induces ECM accumulation and MFT (172).

Taken together, these findings suggests that besides their tolerogenic role, DCs may also play a detrimental role in IPF disease progression. However, due to a lack of convincing evidence on immune regulation by DCs in IPF, their role remains unclear and needs to be studied in more detail.

### Myeloid-Derived Suppressor Cells

MDSCs are immature myeloid-derived cells that potently suppress the immune response, and are mainly subdivided into PMN- and monocytic (M)- MDSCs (173). Due to MDSCs plasticity they have also been found to differentiate into M2-like macrophages and tolerogenic-like DCs (174). The role of MDSCs in cancer is well established, however, their role in many other diseases, such as IPF, remains incompletely defined (14). Increased numbers of PMN-MDSCs were found in the lungs of BPF (and clodronate-treated) mice, likely through increased CXCR 2 expression (40). PMN-MDSCs were also increased in the peripheral blood of patients with interstitial pulmonary disease, while M-MDSC numbers remained unaltered (175). Increased numbers of CD33^+^CD11b^+^ cells (suggestive of MDSCs, particularly M-MDSCs) were found in the peripheral blood in the lungs of IPF patients (69). Increased MDSC numbers were suggested to correlate to poor lung function and increased number of Tregs in IPF patients (69). Depletion of MDSCs was found to enhance fibrosis markers in both kidney and liver models of fibrosis in mice (176), while adoptively transferred MDSCs ameliorated renal fibrosis modeled in mice (177), suggesting a protective role. Increased number of PMN-MDSCs were suggested to be correlated to a decrease in parenchymal fibrosis and attenuation of BPF in mice (175). Another study reported a population of circulating MDSC-like fibrocytes in cancer (178). Furthermore, cells generated from CD11b^+^CD115^+^Gr1^+^ MDSCs were shown to contribute to renal deposition of collagen type I in a murine model of renal fibrosis (179). As mentioned above, PMN-MDSCs have been found to promote Bregs, which was suggested to promote the potential protective role MDSCs play in IPF (133). Bone marrow-derived MSCs have been suggested to drive the differentiation of Gr1^high^CD11b^+^ cells (mainly PMN-MDSCs) towards a Gr1^low^CD11b^+^ phenotype (indicative of M-MDSCs), which was found to inhibit BPF progression in mice (180).

In summary, literature suggests that the immune regulatory functions of anti-inflammatory MDSCs may promote anti-fibrotic effects, making them potentially interesting cells to target in the treatment of IPF. However, there remains a lot of uncertainty on their role, partly attributed to MDSCs plasticity, and more research is needed to further clarify their significance in IPF.

## Interaction of Pharmacological Therapy and Immune Responses in IPF

Previously, immune suppressive medications including glucocorticoids and azathioprine or cyclophosphamide were used in the treatment of IPF (181). In 2015, a new guideline for treatment of IPF formulated strong recommendations against the use of immunosuppressive drugs in IPF (182). This suggestion was primarily based on a single multicenter study, PANTHER-IPF, which was terminated prematurely due to the major safety concerns in patients receiving combination therapy of prednisone, azathioprine, and N-acetylcysteine compared with placebo (183). However, the question remained whether immunosuppressants should be tested once more in combination with anti-fibrotics like nintedanib and pirfenidone in the context of IPF. Further long-term studies are needed to determine the safety and efficacy of the combination of immunosuppressive and anti-fibrotic therapy in IPF.

### Pirfenidone

Pirfenidone is an anti-fibrotic, anti-inflammatory, and antioxidant drug that has been found to interfere with fibroblast proliferation and MFT and the synthesis of ECM (184, 185). Three double blind randomized placebo-controlled Phase III trials (CAPACITY 1 and 2 and ASCEND) approved the efficacy and safety of pirfenidone in PF (186, 187). These studies showed that pirfenidone reduced the rate of decline in forced vital capacity (FVC) over a period of 1 year, by approximately 50%, in IPF patients with mild, moderate and severe impairment in lung function (187, 188). Pirfenidone improved life expectancy in IPF patients, however, its use is unfortunately not associated with significant improvement in clinical symptoms like cough or shortness of breath (187). Pirfenidone has been shown to inhibit pro-inflammatory and pro-fibrotic cytokine production such as TGF-β, IL-4, IL-13, and TNF-α and promotes the production of anti-inflammatory IL-10 (189–194). The beneficial effect of pirfenidone in IPF may indeed be the result of the modulation of immune responses through modulating cytokines. However, the direct modulatory effects of pirfenidone on immune responses were not described for a long time. The direct immune modulatory properties of pirfenidone were first studied by Visner GA., et al., 2009, in a murine model of cardiac allograft transplantation (195). This study demonstrated the direct inhibitory role of pirfenidone on CD4^+^ and CD8^+^ cell proliferation index *in vivo*, whereas pirfenidone showed no effect on the immunosuppressive properties of Tregs (195). The same group of researchers studied the immunomodulatory role of pirfenidone in a murine model of lung allograft transplantation in 2012 (196). Here they showed that pirfenidone suppressed the activation of lung DCs *in vivo* (196). In this respect, *in vitro* treatment of DCs with pirfenidone reduced the expression of major histocompatibility complex (MHC) class II and costimulatory molecules and impaired DC’s capacity to stimulate T cell activation (196). Considering the prolonged allograft survival and robust inhibitory effects of pirfenidone observed in *in vivo* models, as compared to *in vitro* conditions, it may be postulated that other regulatory immune mechanisms are involved in the immunosuppressive effect of pirfenidone besides its direct T cell inhibitory effects. Du et al. showed that pirfenidone reduced splenic germinal center B-cell and Tfh frequencies and reduced infiltration of M2-like macrophages into the lung as well as TGF-β production in a murine model of chronic GVHD (197). Recent studies attempted to investigate the efficacy of pirfenidone co-administered with standard chemotherapy, in cancer. A recent *in vitro* study suggested the inhibitory effect of pirfenidone on metastasis and immune suppressive capacity of cancer-associated fibroblasts (CAFs) in the tumor microenvironment (TME) through inhibition of expression of programmed death-ligand (PD-L) 1 on CAFs and cancer-promoting cytokines and chemokines secretion like TNF-β and CCL17 (198). The immunomodulatory role of pirfenidone needs to be studied in more detail in the TME of cancer models. Although the Food and Drug Administration (FDA) approved pirfenidone for the treatment of IPF since 2014, there remains a lack of robust evidence on the interaction of pirfenidone and the immune system in IPF. Considering the important role of immune responses in IPF, the interaction between pirfenidone and the immune system needs to be studied in more detail, especially in IPF patients receiving long-term pirfenidone treatment.

### Nintedanib

Nintedanib is a triple tyrosine kinase inhibitor targeting PDGF, vascular endothelial growth factor and FGF receptors as well as the non-receptor members of the Src family (199, 200). Nintedanib is an anti-fibrotic and anti-inflammatory drug, and was found to reduce fibroblast proliferation, recruitment, and myofibroblast differentiation, and hinders the secretion of ECM in the lung (199). Two replicate Phase II randomized control studies (INPULSIS-1 and 2) confirmed the safety and tolerability profile of Nintedanib in PF (201). Nintedanib reduced the decline of lung function, improved life expectancy, and reduced the risk of acute exacerbations and mortality (201). Recently, the high affinity of nintedanib to FGF receptors and its favorable toxicity profile resulted in the successful application of nintedanib in combination with conventional chemotherapy in cancer patients, such as second-line therapy for rapidly progressing advanced non-small-cell lung cancer (NSCLC) (202–207). However, the direct impact of nintedanib on the host immune response in the clinical cancer setting as well as in IPF is not completely understood. A recent study showed that nintedanib in combination with paclitaxel, a widely used chemotherapy medication, reduced the number of leukocytes and MDSCs in peripheral blood, and reduced the number of CD8^+^ and B cells in the tumors of Lewis lung carcinoma tumor bearing mice (208). However, nintedanib alone only reduced the mobilization of MDSCs into peripheral blood (208). Overed-Sayer et al., 2020, found that the number of lung mast cells is increased in IPF and was found to be negatively correlated with baseline lung function in humans (209). Additionally, they found that nintedanib, but not pirfenidone, inhibited human fibroblast mediated mast cell survival through stem cell factor receptors and reduced the recruitment of mast cells into the lungs of BPF in rats (209). Although there is a growing body of evidence supporting a dynamic interaction between nintedanib and immune cells, there remains limited understanding on its details, thus warranting further studies.

### Novel Therapies for the Treatment of IPF

Future therapies in IPF would ideally target the phenotype and molecular endotype of IPF patients to further personalize treatments based on guided molecular testing. Completed or still ongoing clinical trials of other pharmacological therapies in IPF are listed (**Table 1**). Although some of the listed medications provided encouraging results on the safety and efficacy in IPF patients (227), several other medications failed to demonstrate benefits in treating patients with IPF in recent clinical trials (213–216). However, many of these new medications may potentially interact with the immune system and be beneficial in some endotype of the IPF patient population but both the exact effects of these medications on immune responses and the beneficial effects remain unclear. In recent years, novel revolutionary anti-cancer agents were also suggested to be used in IPF patients. A recent review article analyzed the available literature on the use of immune checkpoint inhibitors in IPF and suggested only a slight beneficial effect for PD-1/PD-L1 inhibitors (228). Cross talk between PD-1, expressed on CD4^+^ T cells, and PD-L1, expressed on fibroblasts, was proposed to lead to IL17A and TGF-β production by CD4^+^ T cells, which in turn promoted fibrogenesis (228). Recent research also highlighted promising novel drug targets, such as IL-11 in PF, that warrant further studies (229).

**Table 1:** Last completed and/or ongoing clinical trials in idiopathic pulmonary fibrosis.

Drug****	Mechanism****	****Phase	****NCT identifier (48)^3^	Ref.
**Sirolimus (Rapamycin)**	Phosphoinositide 3-kinase (PI3K)/mammalian target of rapamycin (mTOR) inhibitor	N/A	NCT01462006	–
**Omipalisib (GSK2126458)**	PI3K/mTOR inhibitor	I	NCT01725139^*^	(210)
**HEC68498**	PI3K/mTOR inhibitor	I	NCT03502902	–
**Fresolimumab (GC1008)**	Anti-TGF-ß monoclonal antibody	I	NCT00125385^*^	–
**TD139**	Galectin-3 inhibitor	II	NCT02257177^*^/NCT03832946	(211)
**Tipelukast (MN-001)**	Leukotriene antagonist	II	NCT02503657	–
**Belumosudil (KD025)**	Rho-associated coiled-coil kinase 2 (ROCK2) inhibitor	II	NCT02688647	(212)
**CC-90001**	Jun N-terminal kinase inhibitor	II	NCT03142191	–
**Dactrekumab (QAX576)**	Anti-IL-13 monoclonal antibody	II	NCT00532233^*^/NCT01266135^#^	–
**Lebrikizumab**	Anti-IL-13 monoclonal antibody	II	NCT01872689^*^	(213)
**Tralokinumab (CAT354)**	Anti-IL-13 monoclonal antibody	II	NCT02036580^*^/NCT01629667^#^	(214)
**Romilkimab (SAR156597)**	Anti-IgG4 monoclonal antibody that neutralizes IL-4 and IL-13	II	NCT02345070^*^	(215, 216)
**BG00011 (STX-100)^§^**	Anti-integrin (αvβ6) monoclonal antibody	II	NCT01371305^*^/NCT03573505^#^	–
**Simtuzumab (GS-6624)^§^**	Anti-lysyl oxidase like-2 (LOXL2) monoclonal antibody	II	NCT01769196^#^	(217)
**Ianalumab (VAY-736)**	Anti-B-cell activation factor (BAFF) receptor monoclonal antibody	II	NCT03287414	–
**Rituximab**	Anti-CD20 monoclonal antibody	II	NCT01969409/NCT03286556	–
**Carlumab (CNTO 888)^§^**	Anti-CCL2 monoclonal antibody	II	NCT00786201^*^	(218)
**Fezagepras (PBI-4050)**	Ligand of probable G-protein coupled receptor (GPR) 40 and 84	II	NCT02538536^*^	(219)
**BMS-986020**	Autotaxin-Lysophosphatidic acid pathway (ATX-LPA) inhibitor	II	NCT01766817^*^	(220)
**GLPG1690**	ATX-LPA inhibitor	III	NCT03711162/NCT03733444	(221)
**PRM-151**	Pentraxin-2 analogue	III	NCT04552899/NCT04594707	(158, 222)
**ART-123**	Recombinant thrombomodulin	III	NCT02739165^*^	(223)
**Treprostinil**	Prostacyclin	III	NCT04708782	–
**Cotrimoxazole**	Antimicrobial	III	NCT01777737^#^/NCT02759120^#^	(224)
**Doxycycline**	Antimicrobial	III	NCT02759120^#^	(224)
**Sildenafil**	Phosphodiesterase type 5 (PDE5) inhibitor	III	NCT00517933^*^/NCT02802345^*^	(225, 226)
**Pamrevlumab (FG-3019)**	Anti-connective tissue growth factor (CTGF) monoclonal antibody	III	NCT03955146/NCT04419558	(227)

^*^Completed clinical trial.

^#^Clinical trial withdrawn or terminated.

^§^Development in IPF discontinued.

However, our incomplete understanding of IPF pathogenesis, particularly of the role of both structural cells and immune cells in the initiation and progression of IPF, and interaction of current as well as novel therapies with the immune system represent important challenges on the way to success for future precision medicine. To this end, considering the high inter-individual variation in IPF patients, responses to any medication will presumably result in similar variation, thus underscoring the importance of the identification of biomarkers predicting treatment response.

## Immunoregulatory Cells and Lung Transplantation in IPF

Progressive fibrosis in IPF can ultimately result in respiratory failure and death. Here, lung transplantation represents the only therapeutic option that has been linked with a survival benefit in patients suffering from IPF (230). In 2018, a total of 2562 lung transplants were performed in the US, with >60% for underlying disease of IPF (231). However, persisting donor organ scarcity is still limiting the availability of this life-saving therapeutic option. As a result, single-lung transplants (SLT) might represent an effective strategy to serve the increasing demand on donor organs. However, recent analysis of >9.000 patients undergoing lung transplantation for IPF within the United Network of Organ Sharing (UNOS) area demonstrated that double-lung transplantations (DLTs) have improved survival compared to SLTs in patients suffering from IPF (232). Still, five-year survival rates after lung transplantation for IPF are worse compared to other indications (233) with chronic lung allograft dysfunction (CLAD) affecting more than 50% of transplanted lungs after 5 years. Of note, fibrotic processes in the engrafted lung exhibit striking similarities to those in IPF (234).

Since the first report that Tregs are involved in preventing allograft rejection two decades ago (235), immunoregulatory cells have also been widely reported to play a significant role in the context of transplantation and graft tolerance. In more detail, graft acceptance could be linked to complex immunological cross-talk between many more cells, including Bregs, regulatory DCs and especially MDSCs. For example, MDSCs were shown to inhibit GVHD after cell transplantation *via* an arginase-1–dependent mechanism in an experimental model (236). In the clinical context, 50 patients with biopsy-proven acute T cell-mediated rejection (ATCMR) showed increased frequencies of MDSCs in peripheral blood mononuclear cells (PBMC) after renal transplantation, which were linked to improved allograft function compared with the MDSCs low group (237). In addition, MDSCs isolated from 29 adult kidney transplant recipients were demonstrated to expand Tregs *in vitro*, while their accumulation overtime after transplantation linearly correlated with an increase of Tregs *in vivo*, thus providing further evidence for an interactive cross-talk between regulatory cell types (238).

Immunoregulatory cells have also entered the stage in the context of both experimental and clinical lung transplantation. Experimental data demonstrated that long-term lung acceptance is associated with the induction of bronchus-associated lymphoid tissue (BALT), where Tregs accumulate and recipient T cells interact with CD11c^+^ DCs, ultimately resulting in an immune quiescent state (239). Mechanistically, the depletion of Tregs from the BALT of tolerant lungs was shown to result in antibody‐mediated rejection, which was characterized by the generation of donor‐specific antibodies, complement deposition, and the destruction of airway epithelium. In contrast, the undepleted control group showed no evidence of rejection (240). Of note, a recent prospective human observational study could demonstrate that increased Treg frequencies after lung transplantations were reciprocal associated with chronic lung allograft dysfunction (241). Of particular interest, monocyte‐derived DCs, isolated from the peripheral blood of lung transplant patients without bronchiolitis obliterans syndrome, were found to have higher IDO expression, implicating the involvement of MDSCs (242). A recent clinical study focused on MDSCs, assessing their phenotype and frequency in peripheral blood from 20 lung transplant recipients and its relationship to post-transplant complications and immunosuppression (243). In detail, MDSCs were isolated from PBMCs and their functionality was assessed *in vitro* by their capability to block CD4^+^ and CD8^+^ T cell proliferation. As a result, MDSCs were increased in stable lung transplant recipients (n=6) vs. non-transplant controls (n=4). Furthermore, patients with infection (n=5) or CLAD (n=9) had lower MDSCs compared to stable recipients. Of note, MDSCs tended to correlate with blood levels of immunosuppressive medication (i.e. cyclosporine A and tacrolimus).

In addition, Bregs have also been shown to interact during the immunological cross-talk subsequent to allotransplantation by mainly promoting the development of Tregs while at the same time suppressing effector CD4^+^ and CD8^+^ T cells, as demonstrated in experimental models (244). The first clinical study in 117 lung transplant recipients recently revealed that CD19+CD24^high^CD38^high^ Breg cells were associated with chronic rejection while no significant correlation with Tregs was found (245).

Interestingly, a recent publication could demonstrate that the application of MSC–based therapy during *ex vivo* lung perfusion (EVLP) before lung transplantation ameliorated ischemic injury in an experimental pig model. In detail, the MSC group showed significantly lower IL-18 and IFN-γ levels and a significantly higher IL-4 level in lung tissue at 12 hours of EVLP compared to the control group thus reflecting a shift of the inflammatory network towards protective conditions. Of critical relevance, the pathological acute lung injury score after transplantation was significantly lower in the MSC-treated group compared to the control group (246). These experimental data are intriguing and might already point towards future directions where immunoregulatory cells might represent promising candidates for cell therapies.

Thus, there is robust and growing evidence that immunoregulatory cells play a pivotal role in allograft acceptance from both experimental and clinical science (247). Coming back to MDSCs, current data attribute the immunoregulatory properties of these cells mainly to two mechanisms of action upstream of T cells to shift the immune response towards peripheral tolerance: First, to their capacity to inhibit the proliferation of allogeneic T cells (243); Second, to their capacity of Treg induction (248, 249). Interestingly, the impact of concomitant immunosuppressive therapy, to prevent allograft rejection, while at the same time acting on the immune cells in general, and MDSCs in particular, has also become a field of increasing research activities. Here, cyclosporine A, a widely used immunosuppressive drug from the group of calcineurin inhibitors, could be mechanistically linked to increased expression of IDO resulting in increased suppressive activities of MDSCs in a mouse skin transplant model (250).

Tacrolimus, another calcineurin inhibitor used to maintain immunosuppression, has recently been shown to increase the immunosuppressive capacity of MDSCs derived from human kidney allograft recipients *in vitro* (251). In the context of experimental trachea transplantation, the impact of Rapamycin combined with immature DCs (Rapa-imDC), isolated from bone marrow, was investigated in trachea recipient rats receiving Rapa-imDCs for 10 consecutive days after transplantation. Here, Rapa-imDC treatment induced T cell hyporesponsiveness by attenuating T cell differentiation into IFN-γ-producing Th1 cells while at the same time increasing Tregs. In addition, Rapa-imDC administration ameliorated airway obliteration symptoms and CD4^+^ and CD8^+^ T cell infiltration (252).

Taken together, immunoregulatory cells have been shown to play a central role in mediating the alloimmune response towards graft tolerance, thus becoming a promising target for new pharmacologic strategies in the clinical context of transplantation. However, our current understanding of how immunoregulatory cells are interacting in the clinical context of lung transplantation and IPF is still at its infancy, as particular data are missing thus far. Furthermore, we still have to foster our current understanding of how these cells are shaping the alloimmune response while interacting with current immunosuppressive drugs. Here, the clinical background of IPF patients undergoing lung transplantation with poor prognosis is calling for intensive research activities while the concept of tailored cell therapy is already arising from experimental science.

## Conclusion

IPF is the most devastating interstitial lung disease, yet remains poorly characterized and understood. Lack of mechanistic understanding of the complex disease causality and the devastating chronic nature complicate research and development. There remains a lack of efficacy of pharmaceutical therapies and lung transplantation is currently still the only truly effective treatment IPF. Despite recent advances in our understanding of IPF, particularly the role of regulatory immune cells remains contradictory and poorly understood. Indeed most, if not all, of the regulatory immune cells involved exhibit both detrimental as well as beneficial effects in IPF. Substantial evidence underlines the negative role of macrophages in IPF, through the involvement in the dysregulated wound healing process and ECM build-up. On the other hand, the suppressed anti-fibrotic effects observed in lung macrophages hint to potential beneficial effects, resulting in a complex and controversially-discussed role for macrophages in IPF. There are several lines of evidence on the pro-fibrotic role of MSCs in IPF, mainly related to myofibroblast differentiation and cellular senescence. However, recent research on MSCs points to a beneficial role in IPF, mainly related to their anti-inflammatory characteristics. Tregs were also found to act beneficial or detrimental in IPF models, likely influenced by the state of the disease or the disease model used. The role of more recently discovered regulatory immune cells, namely Bregs, tolerogenic DCs, MDSCs and novel macrophage phenotypes in IPF remains unclear as well and needs to be studied in more detail. An increased understanding of the potential role of regulatory immune cells in IPF mouse models and, particularly, in human IPF will be of vital importance to design novel effective pharmaceutical treatments as well as improving the success of lung transplantation and to prevent related GVHD. This is especially true with regards to precision medicine that might define future healthcare frameworks. Therefore, further preclinical and human studies are crucial to better understand and define the role of immune regulatory cells in IPF pathogenesis.

## Author Contributions

SK conceptualized the review. CG, AD, MQ, and SK contributed to the original draft. CG, AD, MQ, HR, DH, and SK contributed to revising and final approval of the manuscript. All authors contributed to the article and approved the submitted version.

## Funding

This work was supported by the fortüne program of the University of Tübingen to MQ (2461-0-0) and SK (2458-0-0 and # 2606-0-0). We acknowledge support by Open Access Publishing Fund of University of Tübingen.

## Conflict of Interest

Author DH was employed by the company Novartis.

The remaining authors declare that the research was conducted in the absence of any commercial or financial relationships that could be construed as a potential conflict of interest.

## Footnotes

1
https://www.clinicaltrials.gov/

2
https://www.clinicaltrials.gov/

3
https://www.clinicaltrials.gov/
